# Logwood as an Antiseptic

**Published:** 1862-10

**Authors:** 


### LOGWOOD AS AN ANTISEPTIC.

IN the present number of the Register, on page 461, Logwood is referred to as an agent, possessing valuable antiseptic properties. Upon reading the extract, the suggestion arises in the mind that it may be valuable, in proper form, as a mouth wash, where there is offensive breath, arising from disease of the teeth or gums; it would certainly be as efficient for this purpose as in the cases referred to.

We shall make some experiments with it, and hope others will do the same. The extract of Logwood is obtainable at any drug store, and is very cheap, costing about twenty-five cents a pound.

